# Patterns of Engagement With the mHealth Component of a Sexual and Reproductive Health Risk Reduction Intervention for Young People With Depression: Latent Trajectory Analysis

**DOI:** 10.2196/70219

**Published:** 2025-07-11

**Authors:** Lydia A Shrier, Carly E Milliren, Brittany Ciriello, Madison M O'Connell, Sion Kim Harris

**Affiliations:** 1 Division of Adolescent/Young Adult Medicine, Boston Children’s Hospital, 300 Longwood Avenue, Boston, MA, 02115, United States, 1 617-355-8306; 2 Department of Pediatrics, Harvard Medical School, Boston, MA, United States; 3 Biostatistics and Research Design Center, Boston Children's Hospital, Boston, MA, United States

**Keywords:** mHealth, ecological momentary assessment, engagement, depressive symptoms, intervention

## Abstract

**Background:**

Mobile health (mHealth) interventions are increasingly used to reduce risk and promote health in real-time, real-life contexts. Engagement is critical for effectiveness of mHealth interventions but may be challenging for young people experiencing depressive symptoms.

**Objective:**

We examined engagement with the 4-week mHealth component of a counseling-plus-mHealth intervention to reduce sexual and reproductive health (SRH) risk among young people with depression (Momentary Affect Regulation – Safer Sex Intervention [MARSSI]) to determine (1) mHealth engagement patterns over time and (2) how sociodemographic characteristics, SRH risks, and depressive symptom severity were associated with these engagement patterns.

**Methods:**

We undertook secondary analysis of data collected from June 2021 to September 2023 in a randomized controlled trial of MARSSI versus a breast health podcast. Eligibility included age 16‐21 years, ability to become pregnant, smartphone ownership, English fluency, past-3-month penile-vaginal sex ≥1x/week and ≥1 SRH risk, and Patient Health Questionnaire-8 item score ≥8. Intervention participants received one-on-one telehealth counseling and then used an app for 4 weeks, responding to surveys (3 prompted at quasi-random, 1 scheduled daily) about affect, effective contraception and condom use self-efficacy, sexual and pregnancy desire, and recent sex, and receiving tailored messages reinforcing the counseling. We computed mHealth engagement days (responding to ≥1 app survey) by week and overall. Latent trajectory analysis identified engagement patterns over the 4 mHealth weeks among participants with any engagement. Using regression analysis, we examined the associations of sociodemographic characteristics, SRH risks, and depressive symptom severity with mHealth engagement patterns and examined moderation by depressive symptom severity. Of the 201 intervention participants, 194 (96.5%) enrolled in the app.

**Results:**

Among those responding to app surveys (167/194, 86.1%), the median engagement was 14 (IQR 4-23) days; 32.9% (55/167) responded on ≥20 days. Overall app engagement (median) declined from 5 (IQR 3-7) days in week 1 to 1 (IQR 0‐5) day in week 4. On latent trajectory analysis, 4 patterns of app engagement emerged: high-throughout (48/167, 28.7%), high-then-declining (40/167, 23.9%), mid-then-declining (47/167, 28.1%), and low-throughout (33/167, 19.7%). Participants identifying gender other than female and those perceiving higher socioeconomic status were more likely to have high-throughout or high-then-declining engagement. Asian or Black non-Hispanic participants and those using low-effectiveness contraception were more likely to have no engagement. In the multivariable model, Asian (adjusted odds ratio [AOR] 0.28, 95% CI 0.10-0.81), Black non-Hispanic (AOR 0.28, 95% CI 0.12-0.66), and higher perceived socioeconomic status (AOR 1.24, 95% CI 1.05-1.48) remained significantly associated with engagement. Engagement patterns showed no differences by depressive symptom severity and no significant moderation.

**Conclusions:**

Young people with depressive symptoms showed initial high engagement with the intervention’s mHealth app to reduce adverse SRH outcomes. Methods to increase and sustain mHealth engagement and differences in engagement by sociodemographic characteristics warrant further studies to optimize the reach of mHealth interventions.

## Introduction

Adolescents and young adults have high rates of unintended pregnancy and sexually transmitted infections (STIs) compared to other age groups [1,2]. Adolescents and young adults who have depressive symptoms are at particularly high risk of these adverse sexual and reproductive health (SRH) outcomes. Although depressed young women may have difficulty forming and sustaining intimate relationships and experience less interest in sexual intercourse, they may have sexual intercourse to manage their depressive symptoms, often under the influence of substances [3] and without condoms or hormonal contraception [4]. These functional encounters may lead to lower self-esteem and bad mood [4]. As a result, depressive symptoms and SRH risk may be self-reinforcing and difficult to change.

The observed associations are consistent with a functional model of sexual behavior, in which motives for sexual behavior can be understood according to motivational (approach and avoidance) and social (intrapersonal and interpersonal) dimensions [5]. Efforts to regulate affect in depression through sexual behavior may aim to enhance positive affect, reduce or cope with negative affect, or both. Individuals with depressive symptoms may also seek validation and affirmation through social interactions. Thus, sexual behavior in depression must be understood in the contexts of both individual affect regulation motives and interpersonal relationships and that these contexts and related experiences influence future motives [6].

The bidirectional, longitudinal relationship of depressive symptoms with sexual behavior and SRH risk is dynamic and can unfold on a moment-to-moment basis. For example, among adolescents and young adults who are depressed, momentary positive affect is associated with having sex [7] and momentary negative affect is associated with having sex with a non–main partner [8]. Furthermore, for adolescents and young adults with depressive symptoms, sexual intercourse events may occur on impulse, without planning [4], and greater impulsiveness is associated with incorrect condom use, if a condom is used [9]. Interrupting the cycle of poor mental health and SRH risk behavior as it develops moment-by-moment in the natural environment may reduce unplanned pregnancy and STIs for these at-risk youth.

Mobile health (mHealth) interventions are well-suited to address the interplay between poor mental health and SRH risk behaviors. Although the definition is subject to some debate [10], in general, mHealth interventions use mobile devices, including phones, tablets, wearable sensors, communication technologies, and other wireless devices, to improve health. As described in several reviews and meta-analyses, mHealth interventions have been applied to the treatment of psychiatric disorders (eg, as a way to strengthen the effects of counseling [11]), contraceptive use [12,13], STI prevention [14], and other SRH outcomes [15]. One form of an mHealth intervention, ecological momentary intervention (EMI) [16], delivers treatment in real-time, real-life contexts, which can be especially helpful for addressing momentary dynamics such as those observed between affective states, substance use, impulsiveness, and SRH risk [4,8,9,17].

To receive mHealth interventions, participants must engage with the mobile device platform, which requires carrying their device, having it powered and on, and with active interventions in which participants provide survey data, responding to queries or initiating reporting. Thus, if participants do not engage with and therefore receive an intervention, the intervention’s effect cannot be assessed [18]. Furthermore, there is a presumption of a dose-response or threshold relationship, such that higher engagement is associated with improved outcomes [19] or that sufficient engagement is required to achieve the intended outcomes (ie, effective engagement) [20].

For individuals with depression, intervention engagement may be difficult to sustain owing to symptoms such as low motivation, poor organization, low energy, behavioral avoidance, negative cognition, difficulty concentrating, and impaired information processing [21-23]. Individuals with psychiatric disorders (eg, schizophrenia) [24] have demonstrated engagement with mHealth interventions focused on mental health [25-27]. However, engagement rates are highly variable and may decline over time for mHealth interventions delivered for weeks to months [24,25,28]. Additionally, individual demographic characteristics and illness severity may be associated with varying levels of engagement with an mHealth intervention [24,27]. Limited information is known about depressed persons’ engagement with mHealth interventions for SRH risk [28]. To fully evaluate an mHealth intervention for adolescents and young adults with the dual challenges of depressive symptoms and SRH risk [28], we must understand engagement with the intervention and the differences in engagement by the level of depressive symptoms and other characteristics of participants from this unique population.

We developed the Momentary Affect Regulation – Safer Sex Intervention (MARSSI) to reduce the risk for unplanned pregnancy and STIs in at-risk adolescents and young adults with depressive symptoms. MARSSI is comprised of a one-on-one main counseling session, 4 weeks of EMI, and a one-on-one booster counseling session at the end of EMI. The counseling uses motivational interviewing to support behavior change and teaches skills from cognitive behavioral therapy for depression. EMI extends the effects of counseling by assessing in-the-moment SRH risk, and if risk is reported, providing personalized, tailored messages to prompt healthy behaviors and encourage cognitive behavioral skill use. We sought to determine (1) patterns of engagement with the 4-week mHealth component of MARSSI and (2) the level of depressive symptoms, SRH risks, and sociodemographic characteristics associated with the engagement patterns.

## Methods

### Ethical Considerations

The parent study (Catherine Henley, PhD, MPH, et al. unpublished data, October. 2024) received institutional review board approval from Sterling institutional review board (approval 8687). All participants provided informed consent. The original institutional review board approval covered secondary analysis without additional consent.

### Participants and Setting

We undertook a secondary analysis of anonymized data collected from June 2021 to September 2023 in a randomized controlled trial of MARSSI versus a breast health podcast (ClinicalTrials.gov NCT04798248). Participants were aged 16‐21 years; biologically able to become pregnant; patients of an SRH care provider, with a visit in the past 2 years; a smartphone owner; fluent in English; not pregnant, desiring pregnancy, or within 6 months post partum; not married or engaged to be married; reporting penile-vaginal sex at least once a week, on average; endorsing at least one SRH risk in the past 3 months; and reporting elevated depressive symptoms as measured by Patient Health Questionnaire (PHQ)-8 score ≥8 [29]. SRH risks in the past 3 months included more than one sexual partner, inconsistent or no condom use, no contraception or low-effectiveness method (ie, condoms, diaphragm, withdrawal, rhythm) as primary form of contraception, penile-anal sex, sex under the influence of alcohol or other drugs, and treatment of an STI. Participants were remunerated up to US $230 in electronic gift cards for completed study activities.

To recruit participants, we partnered with 9 Planned Parenthood affiliates serving 14 US states: Planned Parenthood Great Northwest, Hawai’i, Alaska, Indiana, Kentucky (includes Washington and Idaho); Planned Parenthood of Wisconsin; Planned Parenthood Southeastern Pennsylvania; Planned Parenthood Gulf Coast (Texas and Louisiana); Planned Parenthood of the St. Louis Region and Southwest Missouri; Planned Parenthood Southwest Ohio Region; Planned Parenthood of Illinois; Planned Parenthood Mar Monte (California); and Planned Parenthood of the Pacific Southwest (California). Active recruitment methods at the Planned Parenthood affiliates included study staff sending SMS text messages and patient portal messages directly to patients through the electronic medical record system and affiliate staff mentioning the study to the patients at their appointments. Passive recruitment methods included hanging posters and distributing flyers, as well as posting information on the affiliate website and study-specific or study-affiliated social media accounts on Google, Instagram, and Facebook. Recruitment materials contained a QR code and link to a web-based self-screener for eligibility. In the trial, 405 individuals were enrolled and randomly assigned 1:1 to MARSSI (n=201) versus the podcast (n=204). The randomization scheme used blocking by study coordinator or intervention counselor and by state to account for the potential differences in the availability, quality, and access to reproductive health care services across states. The study coordinator or intervention counselor staff (n=6) could enroll and, per assigned study condition, deliver the intervention counseling to eligible individuals from any US state. Data from participants assigned to the intervention were included in the analyses reported herein.

### Intervention

Participants assigned to MARSSI met with a study coordinator or an intervention counselor (master’s level) for a one-on-one telehealth counseling session in which they chose the SRH risk behavior that they wished to change, developed a change plan, and discussed other SRH risks in brief [28]. Subsequently, for the mHealth component, participants used a smartphone app (MetricWire) for 4 weeks to respond to surveys (3 prompted at quasi-random times, 1 scheduled each day) about their affective states, effective contraception self-efficacy, condom use self-efficacy, desire to have sex, desire to be pregnant, and recent sexual intercourse, and then receive messages based on their responses. After the initial development, the intervention materials were revised to enhance gender inclusivity by limiting the use of gendered pronouns and including the counselor and the participant sharing their preferences for name and pronouns at the outset of the main counseling session.

### Measures

We defined mHealth app engagement as responding to at least 1 survey (momentary or daily) in a day. Because MARSSI emphasizes autonomy over health behavior decisions, we did not expect or require responding to every app survey prompt. Thus, we did not consider response rate as an accurate indicator of app engagement [24,28]. The eligibility survey included PHQ-8, a self-reported measure of depressive symptoms comprising 8 of the 9 Likert-type items (each scored 0‐3) in the well-validated PHQ-9 [30]. The PHQ-8 excludes the question on thoughts of suicide and therefore is recommended in research studies in which further evaluation (ie, with a clinical interview) may not be possible. The score range for PHQ-8 is 0‐24, and the cutoffs for diagnosis of a depressive disorder and the level of symptom severity are the same as those used for PHQ-9 [30]. Consistent with scoring interpretation guidelines [29], we categorized PHQ-8 scores as mild (8-9), moderate (10-14), and moderately severe or severe (15-24) [15-23,31]. Each SRH risk was assessed on the eligibility survey with a single yes-no item. The baseline survey included age (years), race, ethnicity (select all that apply: American Indian or Alaska Native, Asian, Black or African American, Hispanic or Latino/a, Native Hawaiian or other Pacific Islander, White, some other race or ethnicity), gender identity (female, male, transgender female, transgender male, nonbinary or genderqueer, unsure or questioning, I do not identify as any of these), mother’s highest education (did not finish high school, received a high school diploma or general equivalency diploma, completed some college, finished college, finished graduate school, law school, or medical school, don’t know), and self-perceived socioeconomic status (SES) relative to other people in the United States from 10 (most well off) to 1 (least well off) [32].

### Statistical Analyses

All analyses were performed in SAS (version 9.4; SAS Institute) using an α level of .05. We report mean (SD) or median (IQR) for continuous variables and frequency (%) for categorical variables. We examined engagement rates by week and overall. We used group-based latent trajectory modeling to cluster participants by their level of engagement with the mHealth app over 4 weeks [33]. These models were implemented using Proc Traj (SAS Institute) among the participants who ever engaged with the app to identify and group participants into distinct patterns of engagement over the study period. The best-fitting model with respect to the number of engagement groups and polynomial order was selected using the model with the lowest Akaike Information Criterion. Participants who never engaged with the app during the study period formed a “no engagement” category. The identified engagement trajectory patterns comprised a trajectory group variable with categories that were ordered by the initial level of engagement.

We conducted bivariate analyses to examine the associations of sociodemographic characteristics, SRH risks, and severity of depressive symptoms with the identified patterns of mHealth app engagement by using one-way analysis of variance for continuous variables and chi-square tests for categorical variables. We then used multivariable ordinal logistic regression to examine the associations in an adjusted model and to compute the adjusted odds ratios (AORs) and 95% CIs for all predictor variables (AOR>1 indicates greater odds of being in a higher engagement trajectory, AOR<1 indicates lower odds of being in a higher engagement trajectory and thus greater odds of a lower engagement trajectory). In addition, we explored the potential for the effect modification of depressive symptom severity with sociodemographic characteristics and SRH risks by testing the interaction terms in a multivariable regression model.

## Results

### Baseline Characteristics of the Participants

Of the 201 intervention participants, 194 (96.5%) enrolled in the app and were included in the analyses herein. Participant characteristics at baseline are shown in Table 1.

**Table 1.** Sociodemographic characteristics of the participants using the mobile health app component of the Momentary Affect Regulation – Safer Sex Intervention—overall and by pattern of app engagement.

Characteristic	Engagement pattern						*P* value
	Overall (n=194)	No engagement (n=27)	Low-throughout (n=33)	Mid-then- declining (n=46)	High-then- declining (n=40)	High-throughout (n=48)	
Age (years), mean (SD)	19.1 (1.1)	19.2 (1.1)	19.4 (1.1)	19.1 (1.1)	18.9 (1.1)	19.1 (1.1)	.45
Gender, n (%)							.04
Female	169 (87)	23 (85)	31 (94)	39 (85)	39 (98)	37 (77)	
Other	25 (13)	4 (15)	2 (6)	7 (15)	1 (2)	11 (23)	
Race and ethnicity, n (%)							.006
Asian	14 (7)	4 (15)	4 (12)	1 (2)	4 (10)	1 (2)	
Black or African American, non-Hispanic	22 (11)	8 (29)	5 (15)	3 (7)	3 (7)	3 (6)	
Hispanic or Latino/a	22 (11)	4 (15)	2 (6)	10 (22)	3 (7)	3 (6)	
White, non-Hispanic	94 (49)	10 (37)	17 (52)	19 (41)	21 (53)	27 (56)	
Another race or ethnicity^a^	42 (22)	1 (4)	5 (15)	13 (28)	9 (23)	14 (29)	
Mother’s highest education (n=193), n (%)							.62
Less than college graduate	129 (66)	19 (70)	23 (70)	34 (74)	26 (65)	27 (56)	
College degree or beyond	64 (33)	8 (30)	10 (30)	12 (26)	14 (35)	20 (42)	
Perceived socioeconomic status (1-lowest to 10-highest), mean (SD)	5.0 (1.6)	4.9 (1.6)	4.6 (1.8)	4.8 (1.6)	5.1 (1.5)	5.6 (1.4)	.06
Sexual and reproductive health risk in the past 3 months, n (%)							
Sex without condom	180 (93)	25 (93)	32 (97)	40 (87)	38 (95)	45 (94)	.47
Low-effectiveness contraception as primary method^b^	113 (58)	21 (78)	13 (39)	24 (52)	29 (73)	26 (54)	.009
More than 1 partner	70 (36)	10 (37)	10 (30)	18 (39)	18 (45)	14 (29)	.55
Sex within 2 hours after using alcohol or drugs (n=193)	125 (64)	17 (63)	22 (67)	31 (67)	24 (60)	31 (65)	.68
Treated for an STI^c^	39 (20)	6 (22)	7 (21)	8 (17)	8 (20)	10 (21)	.99
Number of risks, mean (SD)	2.7 (1.1)	2.9 (1.1)	2.5 (1.1)	2.6 (1.1)	2.9 (1.0)	2.6 (1.2)	.42
Depressive symptoms, mean (SD)							
PHQ-8^d^ score (0‐24)	13.0 (4.2)	13.3 (3.6)	13.3 (4.8)	12.1 (3.9)	13.5 (4.9)	13.1 (3.9)	.54
Severity (score range), n (%)							.80
Mild (8-9)	49 (25)	4 (15)	10 (30)	14 (30)	9 (22)	12 (25)	
Moderate (10-14)	84 (43)	15 (55)	11 (33)	20 (44)	18 (45)	20 (42)	
Moderately severe to severe (≥15)	61 (31)	8 (30)	12 (36)	12 (26)	13 (33)	16 (33)	

a Includes American Indian or Alaska Native, Native Hawaiian or other Pacific Islander, other race, and multiple race or ethnicity.

b Used condoms, diaphragm, cervical cap, spermicide, sponge, fertility awareness, or withdrawal as the primary form of contraception.

c STI: sexually transmitted infection.

d PHQ-8: Patient Health Questionnaire-8 items.

### App Engagement

Of the 194 participants, 167 (86.1%) responded to at least one app survey (ie, had at least one day of engagement with the app) and 27 (13.9%) did not respond to any survey during the 4-week study period (no engagement). Among participants who ever engaged with the app, median engagement was 14 (IQR 4‐23) days out of 28 days; 32.9% (55/167) responded on ≥20 days. The overall median engagement among app users declined from 5 days in week 1 to one day in week 4.

**Figure 1.**
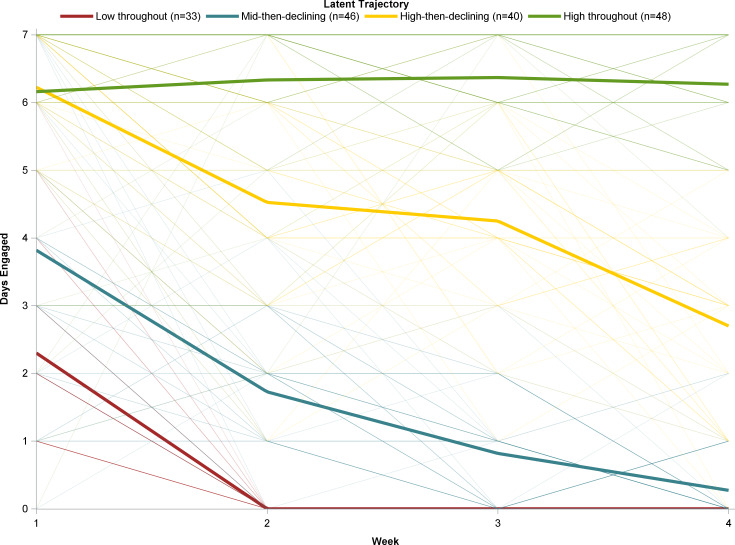
Latent trajectories for days of app engagement across 4 weeks among participants who had any app use in the Momentary Affect Regulation – Safer Sex Intervention.

The latent trajectory analysis of the app users revealed 4 patterns of any engagement across the 4 weeks: high-throughout (55/167, 32.9% of participants with any engagement), high-then-declining (40/167, 23.9%), mid-then-declining (47/167, 28.1%), and low-throughout (33/167, 19.7%) (Figure 1). Participants in the high-throughout trajectory tended to use the app on 6 or 7 days every week for all 4 weeks. The high-then-declining group also used the app nearly every day in week 1 but declined in use each week thereafter to around 3 days in week 4. Participants in the mid-then-declining group began use on about half the days in week 1 and then declined over the weeks to use on 0 or 1 day in week 4. In the low-throughout group, participants engaged with the app only in week 1 and then discontinued use.

### Associations of Sociodemographic Characteristics, Sexual Risk, and Depressive Symptom Severity With App Engagement

In the bivariate analysis (Table 1), there were statistically significant differences in the level of engagement by gender identity (*P*=.04), race and ethnicity (*P*=.006), and report of low-effectiveness contraception as the primary method in last 3 months (*P*=.009). Participants identifying gender other than female were more likely to have high-throughout engagement. Those in the high-then-declining engagement group tended to perceive a higher SES score than other groups. Asian and Black or African American, non-Hispanic participants, and those using low-effectiveness contraception were more likely to have no engagement. There were no differences in the engagement patterns by depressive symptom severity.

**Figure 2.**
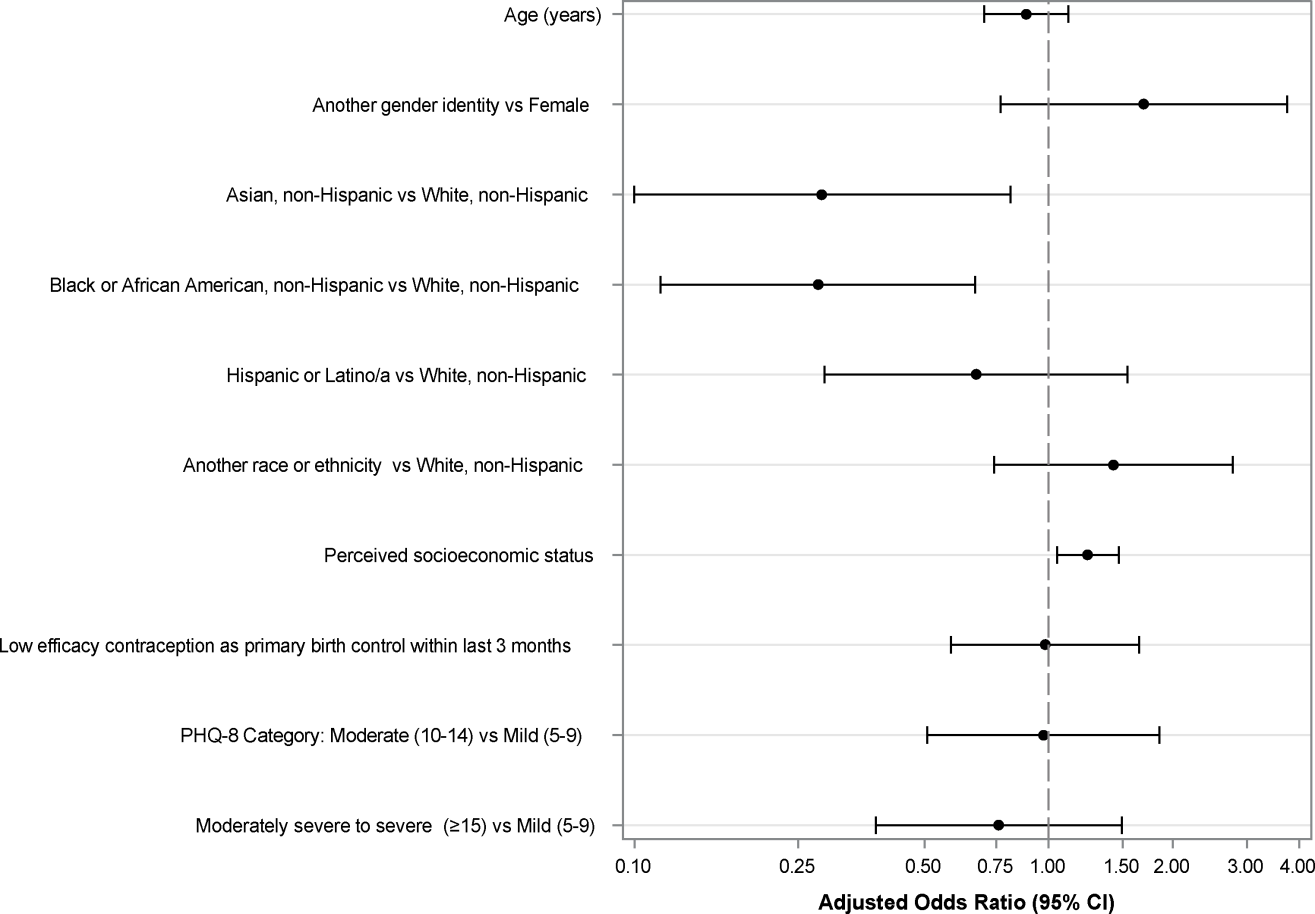
Multivariable ordinal logistic regression model examining associations of sociodemographic characteristics, sexual and reproductive health risk, and depressive symptom severity with trajectory of engagement with the Momentary Affect Regulation – Safer Sex Intervention app. PHQ-8: Patient Health Questionnaire-8 items.

In the multivariable model (Figure 2), race and ethnicity remained significantly associated with lower engagement with participants identifying as Asian (AOR=0.28; 95% CI: 0.10, 0.81) or Black or African American, non-Hispanic (AOR=0.28; 95% CI: 0.12, 0.66) less likely to have higher engagement compared to those identifying as White, non-Hispanic. Higher perceived SES was associated with higher engagement (AOR 1.24, 95% CI 1.05-1.48). Gender identity and use of low-effectiveness contraception were no longer statistically significant after adjustment for other covariates (gender identity: AOR 1.70, 95% CI 0.77-3.76; low-effectiveness contraception: AOR 0.98, 95% CI 0.58-1.65). The severity of the depressive symptoms was not associated with the engagement pattern and did not moderate associations between the sociodemographic characteristics and the level of engagement.

## Discussion

### Main Findings

This study investigates the patterns of mHealth engagement among adolescents and young adults with depressive symptoms assigned to a counseling-plus-mHealth intervention to reduce their risk for unintended pregnancy and STIs. Despite all adolescents and young adults in the study having at least mild depressive symptoms, most participants engaged with the 4-week mHealth component at least once, one-half of the participants engaged on at least 50% of the days, and one-third of the participants engaged on more than 70% of the days. Importantly, for MARSSI, the severity of the depressive symptoms did not appear to influence engagement. Women with depression may benefit from risk reduction interventions to a degree similar to women without depression [34,35]. Additionally, interventions may be more effective in reducing the risk for young women with depression if they address factors related to emotional distress [36-38]. The results of our study add evidence that mHealth interventions to reduce SRH risk that include strategies to manage psychiatric symptoms engage participants, and thus, the effectiveness of these types of interventions can be evaluated across the full range of depressive symptoms.

Across all participants with any mHealth engagement, engagement declined substantially over 4 weeks, as seen with engagement in other longitudinal studies of mHealth interventions [24,39]. However, using latent trajectory analysis, we identified 4 distinct patterns of engagement among those with any use of the mHealth app, demonstrating variability in use over time that was not evident in the overall analysis. More than one-fourth of the participants with any app engagement sustained their engagement for all 4 weeks, and another nearly one-fourth had high initial engagement that declined but remained moderate (on about half of days) through week 4 of EMI; in total, these participants represented nearly one-half of all the participants who enrolled in the app, including those who did not engage at all. Thus, overall declining engagement with an mHealth app may still be associated with continued intervention exposure for a substantial proportion of participants. Furthermore, declining engagement may be a desirable or an undesirable effect in a multiweek mHealth intervention [40]. For example, participants may have decreased app use as a result of habituation or accumulated burden—an undesirable effect of repeated app use. However, declining engagement may have been a function of perceived or actual intervention effect, a desirable consequence of app exposure; in other words, if a participant experiences improvement, that person may determine that the app is not needed as much or at all and elect to engage with it less or discontinue use altogether. Future research will need to determine the thresholds for intervention effectiveness and the reasons for app disengagement. mHealth studies should take an expansive approach to evaluating app engagement, including both querying participants regarding their reasons for app use or nonuse and examining participant characteristics for associations with engagement.

In this study, we found differences in engagement over time by sociodemographic characteristics. First, participants identifying as Asian or as Black or African American, non-Hispanic, compared to those identifying as non-Hispanic White tended to have lower engagement trajectories. Other studies of mHealth interventions for behavior change [41] and in individuals with psychiatric disorders have found lower app engagement among participants from minoritized racial and ethnic groups, compared to White participants [24,27,42]. Smartphone usage patterns and dependency for online access differ among racial and ethnic groups [43,44]. For example, compared to White teens, Black teens report higher rates of using most common social media platforms and of being on social media almost constantly [43], and Black and Asian adults report higher rates of smartphone dependency compared to White adults [44]. Participants who identified as Asian or as Black may have been lesser engaged with the study app than White participants because they were more likely to have been using their smartphones for other purposes such as accessing social media sites. However, individuals from minoritized racial and ethnic groups have also demonstrated lower engagement with in-person interventions [24,42], suggesting that there may be alternative or additional explanations for the observed differences in engagement, such as race as proxy for social determinants of health or racism [45].

Second, we found that intervention participants with higher perceived SES tended to have higher engagement trajectories. Financial resources may also influence engagement with mHealth interventions. Although nearly all adolescents and young adults in the United States have access to or own a smartphone [46], there may be differences in smartphone usage related to SES. Adults with lower household incomes and those with lower levels of formal education are more likely than their peers to be dependent on a smartphone for online access [44] and thus may have less time free from other smartphone activities to attend to the intervention app. As with the associations of engagement with race and ethnicity, other factors may explain the positive correlation between engagement and SES, such as social context, time use, or mobile phone data plan. It will be important to understand and mitigate disparities in app engagement by sociodemographic characteristics to fully evaluate the effectiveness of mHealth interventions across racial, ethnic, and economic groups.

### Limitations

Our findings contribute to the limited literature on mHealth interventions for adolescents and young adults with SRH risk that address emotional distress or depressive symptoms [47], with some caveats. In recruiting participants from across the United States and using both active and passive recruitment methods, this study yields a sample diverse in gender, race, ethnicity, and perceived SES. This diversity permitted analyses into differences in mHealth engagement for groups at particularly high risk, yet underrepresented in mHealth research [48]. However, because there were several distinct engagement patterns, a larger sample was needed to find small differences in engagement by sociodemographic factors that may be important for assessing and improving intervention effectiveness (eg, characteristics of participants who sustained high engagement vs those whose engagement was initially high but then declined). Additionally, engagement was specific to the MARSSI mHealth component, and the patterns and associations may not be generalizable to other interventions. Although MARSSI development employed a human-centered design [28,49], further tailoring of the intervention to address the unique needs of adolescent and young adult participants from minoritized and underresourced groups, rooted in their individual and cultural contexts, may increase their mHealth engagement [50].

### Conclusions

In a counseling-plus-mHealth intervention to reduce SRH risk, young people with depressive symptoms demonstrated varied patterns of engagement over 4 weeks of mobile app use. It will be important to establish what level of engagement with the intervention is required to achieve positive outcomes (ie, constitutes effective engagement) [20]. Patterns of mHealth engagement did not differ by level of depressive symptoms, suggesting that mHealth interventions for SRH risk may be applied in those with and without depressive symptoms. mHealth engagement patterns did differ by racial group and perceived SES. Future research is needed to understand and address disparities in engagement with mHealth interventions as a necessary step toward improving evaluations of effectiveness and expanding reach for this rapidly growing method of intervention delivery [20].

## Abbreviations

AOR: adjusted odds ratio
EMI: ecological momentary intervention
MARSSI: Momentary Affect Regulation – Safer Sex Intervention
mHealth: mobile health
PHQ: Patient Health Questionnaire
SES: socioeconomic status
SRH: sexual and reproductive health
STI: sexually transmitted infection
